# Enhanced adsorptive composite foams for copper (II) removal utilising bio-renewable polyisoprene-functionalised carbon derived from coconut shell waste

**DOI:** 10.1038/s41598-020-80789-x

**Published:** 2021-01-14

**Authors:** Wachiraporn Kettum, Chanatip Samart, Narong Chanlek, Phakkhananan Pakawanit, Prasert Reubroycharoen, Guoqing Guan, Suwadee Kongparakul, Suda Kiatkamjornwong

**Affiliations:** 1 Department of Chemistry, Faculty of Science and Technology, Thammasat University, Pathumthani, 12120 Thailand; 2 Bioenergy and Biochemical Refinery Technology Program, Faculty of Science and Technology, Thammasat University, Pathumthani, 12120 Thailand; 3 Synchrotron Light Research Institute (Public Organization), 111 University Avenue, Muang District, Nakhon Ratchasima, 3000 Thailand; 4 Department of Chemical Technology, Faculty of Science, Chulalongkorn University, 254 Phyathai Road, Wangmai, Patumwan, Bangkok, 10330 Thailand; 5 Institute of Regional Innovation, Hirosaki University, Aomori, 030-0813 Japan; 6 Office of University Research Affairs, Chulalongkorn University, 254 Phyathai Road, Wangmai, Patumwan, Bangkok, 10330 Thailand; 7 FRST, Academy of Science, Office of the Royal Society, Sanam Suea Pa, Khet Dusit, Bangkok, 10300 Thailand

**Keywords:** Soft materials, Nanocomposites

## Abstract

A bio -renewable polyisoprene obtained from *Hevea Brasiliensis* was used to produce functionalised carbon composite foam as an adsorbent for heavy metal ions. Functionalised carbon materials (C-SO_3_H, C-COOH, or C-NH_2_) derived from coconut shell waste were prepared via a hydrothermal treatment. Scanning electron microscopy images showed that the functionalised carbon particles had spherical shapes with rough surfaces. X-ray photoelectron spectroscopy confirmed that the functional groups were successfully functionalised over the carbon surface. The foaming process allowed for the addition of carbon (up to seven parts per hundred of rubber) to the high ammonia natural rubber latex. The composite foams had open pore structures with good dispersion of the functionalised carbon. The foam performance on copper ion adsorption has been investigated with regard to their functional group and adsorption conditions. The carbon foams achieved maximum Cu(II) adsorption at 56.5 ${\text{mg g}}_{\text{foam}}^{-1}$ for C-SO_3_H, 55.7 ${\text{mg g}}_{\text{foam}}^{-1}$ for C-COOH, and 41.9 ${\text{mg g}}_{\text{foam}}^{-1}$ for C-NH_2_, and the adsorption behaviour followed a pseudo-second order kinetics model.

## Introduction

Ecosystems and the environment as a whole are threatened by the continuous heavy metal contamination of natural water sources. A considerable increase in the use of heavy metals in industries such as electroplating, metal finishing, metallurgy, tanneries, chemical manufacturing, mining, and battery manufacturing have led to the release of large amounts of untreated wastewater into the environment^1^. Unlike organic pollutants, heavy metal ions and their compounds are not degradable and have a tendency to accumulate in living beings, often leading to toxic or carcinogenic effects. At high concentrations, Cu(II) has been linked to liver and kidney damage, and increased blood pressure and respiratory rates in humans, while Ni(II) is associated with the lung and kidney disorders, chest pain, and shortness of breath^2,3^. The hexavalent form of chromium is a well-known human carcinogen and can cause liver damage, pulmonary congestion, and skin irritation, which result in ulcer formation^4^. Therefore, these metals must be removed from wastewater before their discharge into natural water bodies, and an effective method for doing so must be developed.

Numerous conventional methods for the removal and recovery of heavy metals from contaminated industrial waste streams have been reported. These processes can be classified into three types: chemical, physical, and biological, where physical and chemical processes are more widely implemented. Chemical methods include flotation, coagulation/flocculation, chemical precipitation, electrochemical processes, and ion exchange. Membrane filtration and adsorption are the most widely used physical methods as they are inexpensive, biodegradable, and effective^1,5^.

Composites of inorganic or organic fillers with natural polymer matrix have received greater attention due to their adsorption ability. Special characteristics such as antibacterial activity by adding Ag nanoparticles, magnetic properties by adding ferroferric oxide particles, and mechanical/thermal resistant property by adding organoclay can be designed. Moreover, various petroleum-based polymers have been used for adsorbent preparation such as poly (ethylene glycol dimethacrylate-*n*-vinyl imidazole) and poly(divinylbenzene-*n*-vinylimidazole)^6–9^ for specific adsorption of metal ions. Since the composites mainly consist of fillers and polymer matrix, the functionalisation of fillers required proper modification to ensure high dispersion. In this study, composites foam of functionalised activated carbon with a natural polymer matrix was selected because they are naturally available.

Activated carbon is a versatile adsorbent owing to an extensive surface area, microporous structure, strong and universal adsorption capacity, and high degree of surface reactivity. Carbon materials have been functionalised with a wide variety of functional groups. Sulfonyl groups (−SO_3_H) can be added using hydrothermal carbonisation and sequential sulfonation to produce carbon with a porous functionalised surface^10–14^. Carbon foam is a popular alternative adsorbent because of its strength, low weight, and low cost and is typically prepared by dip coating polyurethane (PU) with pitch slurry, activated carbon slurry, or metal oxide solution, followed by carbonisation. Three-dimensional porous carbon-based materials have been used for dye adsorption, heavy metal adsorption, and electromagnetic shielding^15–19^. Carbon foam can remove heavy metal ions from aqueous solutions including copper, zinc, cadmium, lead, and nickel^18,19^. Previous reports have investigated the use of a synthetic polymer (e.g. PU foam or phenolic resin-based foam) as a porous support material. To prevent the use of environmentally harmful materials, this study aimed to investigate the formation of functionalised carbon and carbon foam for heavy metal adsorption using a bio-renewable material. Porous carbon obtained from coconut shells was functionalised with sulfonic acid (−SO_3_H), carboxylic acid (−COOH) and amine (−NH_2_) groups. Good dispersion and distribution throughout the foam was required, and the adsorption efficiency was investigated. The effects of the pH of heavy metal solution, contact time, and concentration of the adsorbate on adsorption were evaluated to better understand the adsorption kinetics.

For Cu(II) adsorption, different type of copper complex has been used, for example copper chloride, copper acetate, copper nitrate or copper sulfate. Various techniques, such as, spectrophotometer, atomic adsorption spectroscopy (AAS), and inductively coupled plasma spectrometer (ICP), have been used according to the amount of Cu(II) in aqueous media. Atomic adsorption spectroscopy (AAS) can be applied for Cu(II) concentration range of 5–400 ppm which the measurement by AAS perform higher sensitivity than spectrophotometer^20–23^. Trace amount of Cu(II), such as 5–200 ppm, can be studied by using ICP which perform highest sensitivity compare to spectrophotometer and AAS^24^.

Our research studied Cu(II) adsorption by using spectrophotometer measurement. A similar work using spectrophotometer on Cu(II) adsorption studied with 500 ppm^25^. The permissible limit of Cu(II) in water is less than 2.5 mg L^−1^^26^. Several treatment technologies such as solvent extraction, ion exchange, and precipitation have been suggested and employed to remove heavy metals from aqueous solution, however, these technologies are costly and also create another problem with metal-bearing sludge^27^. Therefore, our research work focused on an economical and available adsorbent for removing copper from aqueous solution by using simple technique such as spectrophotometer.

## Results and discussion

### Characteristics of functionalised carbon and carbon foam

**Figure 1:**
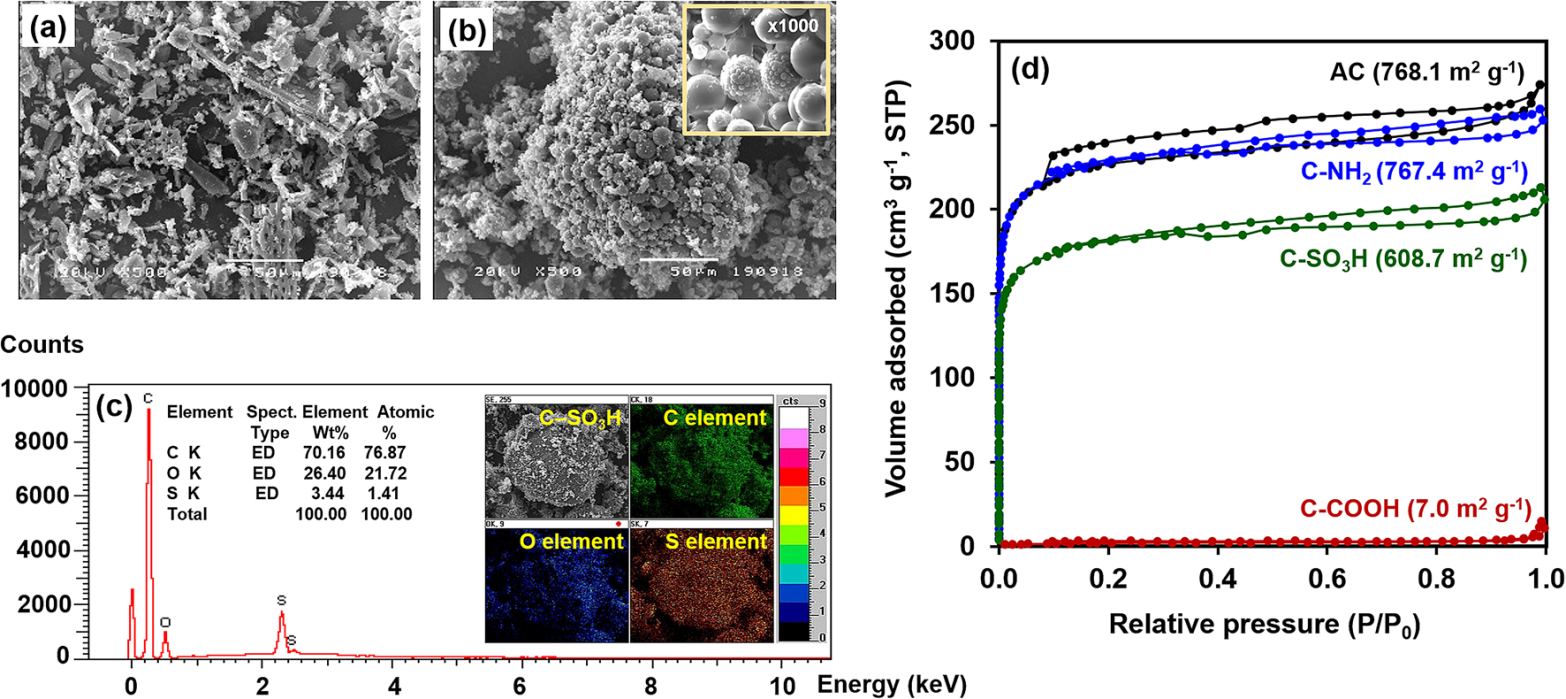
SEM images of **(a)** activated carbon and **(b)** C-SO_3_H (inset at  × 1000 magnification). **(c)** SEM-EDX elemental maps of C-SO_3_H. **(d)** N_2_ adsorption-desorption isotherms of the untreated carbon and functionalised carbon.

The untreated carbon particles have an irregular morphology and appear as partially flattened cylinders, as shown in the SEM images (Fig. 1a). After functionalisation via hydrothermal treatment, the particles become smaller and spherical with a rough surface (Fig. 1b). An example of the C-SO_3_H particles is shown in Fig. 1c, where clarity of elements distribution allows the elemental mapping. The foam surface primarily contains C, but S from the sulfonyl group is evenly distributed across the carbon surface. C-NH_2_ and C-COOH could not be clearly characterised using SEM-EDX, and therefore, XPS was used to evaluate the binding energy of the functionalised carbon. From Fig. 1d, the (BET) surface areas (S_BET_) of the carbon samples decrease after hydrothermal treatment, particularly for the C-COOH samples. This decrease in the surface area is attributed to the structural deformation or collapse of the carbon framework caused by the strong reaction conditions^28^. SEM-EDX was used to characterise the distribution of the functional groups across the carbon surface.

**Figure 2:**
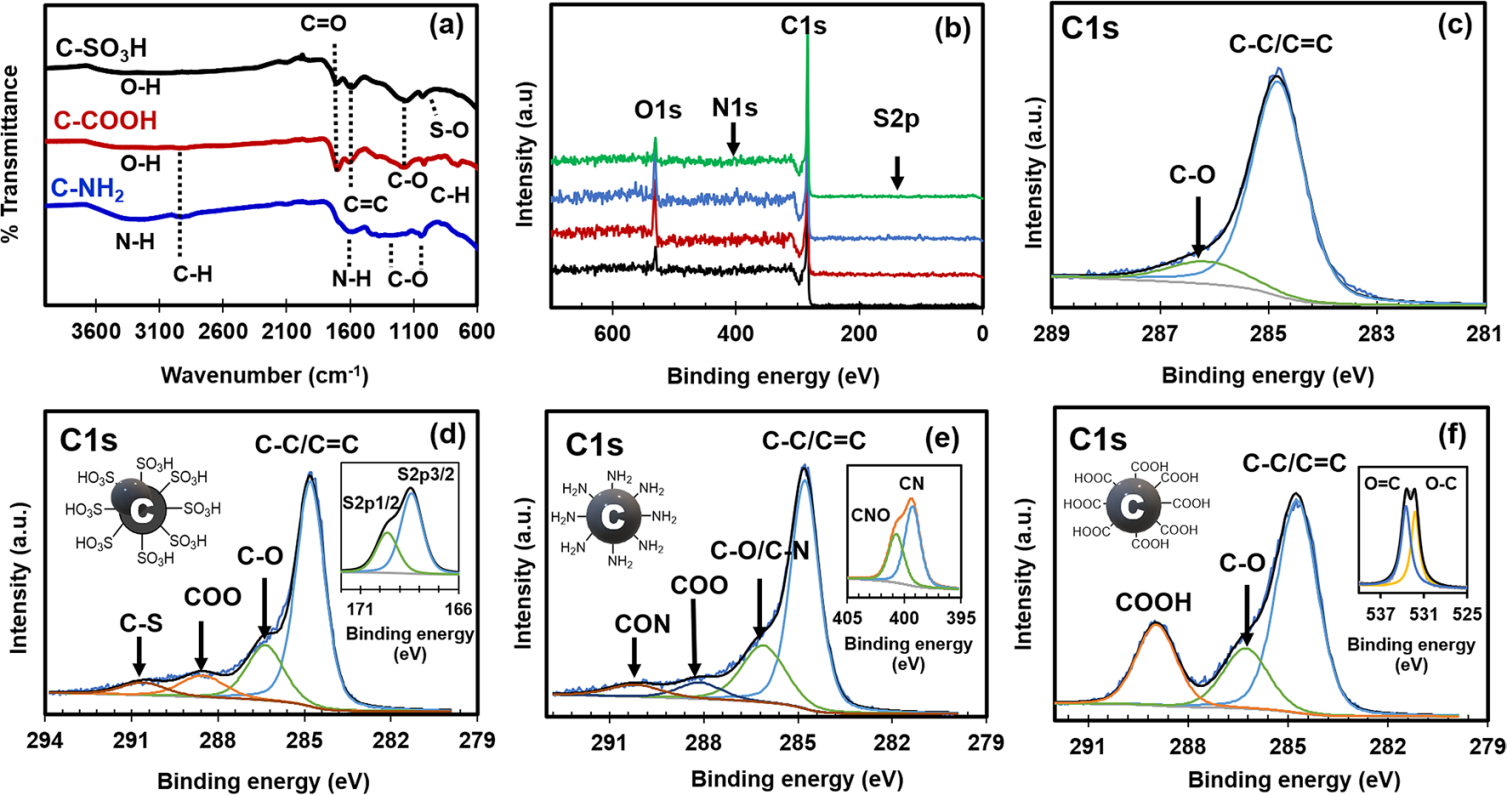
**(a)** FT-IR spectra of functionalised carbon. **(b)** XPS survey spectra and deconvolution of the core-level spectra of C1s of the **(c)** untreated carbon, **(d)** C-SO_3_H (with the inset of S2p), **(e)** C-NH_2_ (with the inset of N1s), and **(f)** C1s of C-COOH (with the inset of O1s), respectively.

The FT-IR spectra of the sulfonated carbon (C-SO_3_H) exhibits the absorption peaks at 1702, 1588, and 1155 cm^−1^ corresponding to C=O, C=C, and C–O bonds, respectively (Fig. 2a). The peak at 1030 cm^−1^ is attributed to the –SO_3_H group. Aromatic C–H bonds and O–H bonds in the carboxylic acid and phenol groups absorb strongly from 3600 to 2400 cm^−1^. The spectra of the carboxylated carbon (C-COOH) is characterised by an adsorption band at 1697 cm^−1^, attributable to the carboxylate group. The signals at 1607 cm^−1^ and 1176 cm^−1^ correspond to C=C and C-OH stretching, respectively, and indicate that the residual hydroxyl groups are present at the hydrophilic surface. The spectra of the carbon modified with ammonia (C-NH_2_) exhibit a broad peak at 3242–3400 cm^−1^, and the peak at 1591 cm^−1^ is attributed to the stretching and bending vibrations of –NH_2_, indicating that the amino groups are present at the surface. The peaks in the 1000–1400 cm^−1^ range correspond to the oxygen-rich groups (C–O) in the amino-functionalised carbon.

XPS was used for the analysis of the surface composition by determining the elemental composition of the material and binding energy of the elements on the surface. The high-resolution XPS survey spectra shown in Fig. 2b–f verify the composition of the samples. Wide-scan survey spectra confirm the presence and atomic concentrations of the elemental C, O, S, and N on the carbon sample surfaces (Table 1). The major elements are C (90.89%) and O (9.11%), and the atomic concentration of O increases in the carboxyl-functionalised carbon samples compared to the untreated samples due to the oxygen content of the –COOH group. Atomic concentration of 0.5% is observed for S in the C-SO_3_H samples, confirming the presence of the –SO_3_H group on the carbon surface. Similarly, the amine-functionalised carbon, C-NH_2_, has an atomic concentration of 3.51% for N in the –NH_2_ group. Details of the functionalised carbon surface were determined from the C1s high-resolution XPS spectra (Fig. 2c–f). In the untreated carbon samples, the peaks corresponding to C are observed at 284.5, 284.9, and 286.8 eV, attributed specifically to sp^2^ C (C=C), sp^3^ C (C–C), and C–O (hydroxyl), respectively. Functionalisation leads to the appearance of new peaks. These include a peak at 289 eV corresponding to the –COO groups in C-COOH, 290 eV correlating to the C–S bond in C-SO_3_H (confirmed by the S2p core level spectrum), and 399 eV attributable to the N1s peak for C–N bonding in C-NH_2_. The C-COOH samples exhibit an increase in the carboxylic acid peak intensity, including O1s of O=C and O–C peaks at 533.3 and 531.9 eV. Similar XPS results have been previously reported in literatures^29–33^.

**Table 1:** Atomic concentrations of the untreated carbon and functionalised carbon samples.

Samples	Atomic concentration (%)			
	C	O	S	N
Activated carbon	90.89	9.11	0	0
C-SO_3_H	83.11	16.39	0.50	0
C-COOH	67.82	32.18	0	0
C-NH_2_	82.47	14.02	0	3.51

**Figure 3:**
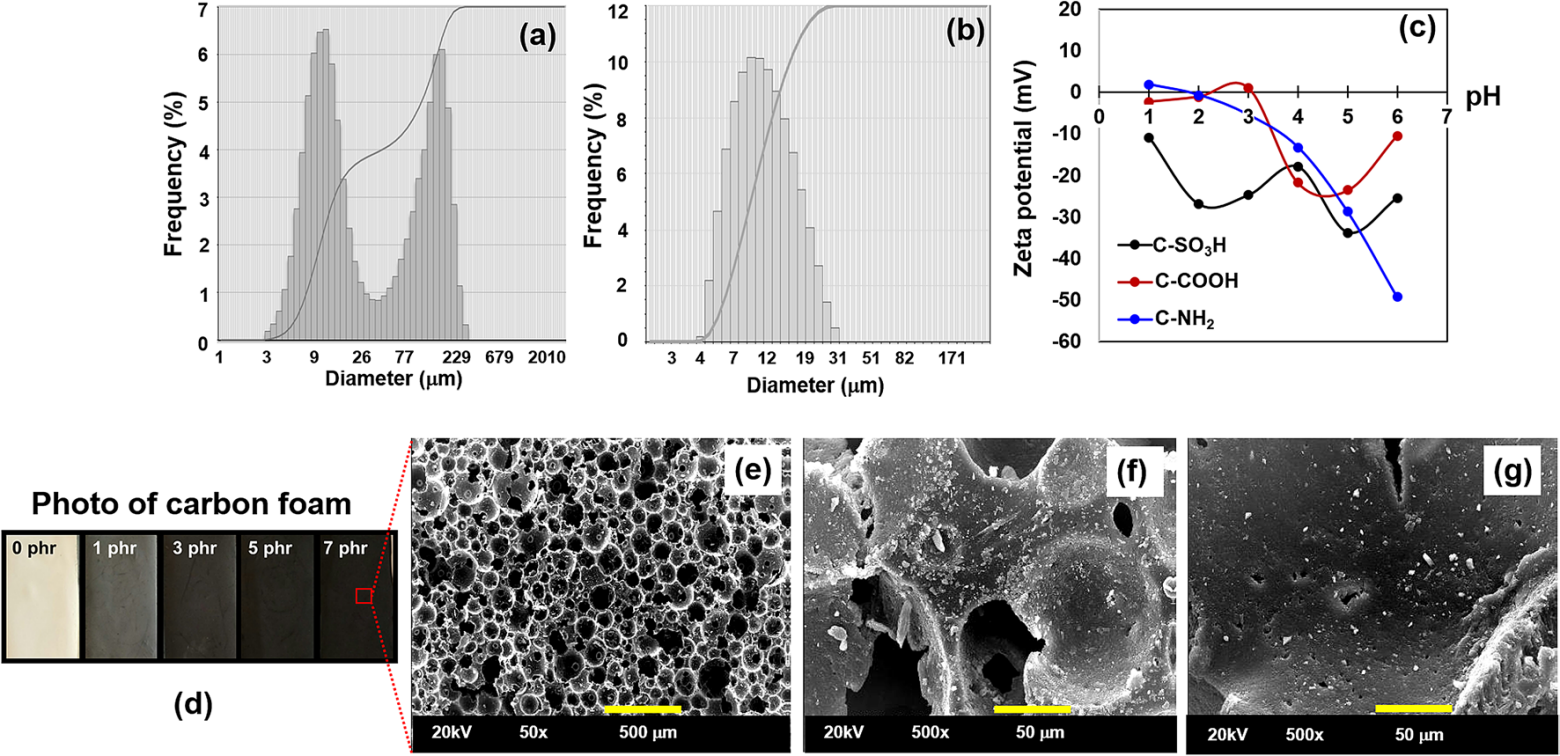
Particle size analysis of **(a)** activated carbon from coconut shell and **(b)** carbon slurry. **(c)** Zeta potential of the functionalised carbon as a function of pH. **(d)** Photographs taken using a CCD camera. SEM images of the **(e)** open cell structure of the carbon foam, **(f)** carbon foam containing unground carbon, and **(g)** carbon foam containing ground carbon.

Functionalised carbon slurries with concentrations ranging from 1 to 7 phr were added to the NRL. Curing agents were used to produce the carbon foam, which was subsequently steam vulcanised to yield a final bio-renewable polyisoprene-functionalised carbon composite foam with an open-cell structure. Typically, a rubber composite with a high carbon content (up to 70 phr) is produced using a conventional two-roll mill^34^. However, NRL is a colloidal dispersion of *cis*-1,4-polyisoprene in an aqueous medium, and the amount of carbon that can be added to the latex is limited due to issues related to miscibility and phase separation of the carbon powder. Nanogrinding methods can be used to reduce the particle size in order to increase the carbon content (Fig. 3a,b). The average particle size of carbon after nanogrinding is 4–31 µm, which is smaller than that of activated carbon (3 to 229 µm). The zeta potential as a function of pH of the functionalised carbon in water suspensions indicates that the points of zero charge (pH_pzc_) of C-NH_2_ and C-COOH are observed at a pH of 2 and 3, respectively, while C-SO_3_H is negatively charged in the pH range of 1–6 (Fig. 3c). These observations are attributed to the effects of the surface functional groups on the suspension behaviour and heavy metal adsorption.

The colour of the carbon foam before and after the addition of carbon slurry is shown in Fig. 3d, which shows that the foam colour darkens with increasing carbon content. The SEM images reveal the open pore structure of the foam, with cell interconnection and fine particles distributed in the open cell wall (Fig. 3e). The open-cell wall of the unground carbon (Fig. 3f) shows agglomeration and non-uniform distribution, while the nanoground carbon slurry (Fig. 3g) has a better distribution in the rubber matrix (up to 7 phr). A pore diameter of below 200 µm is observed for the open pore structure, and the dispersion of carbon across the surface can also affect adsorption.

### Effect of contact time, pH, and initial concentration on Cu(II) adsorption

**Figure 4:**
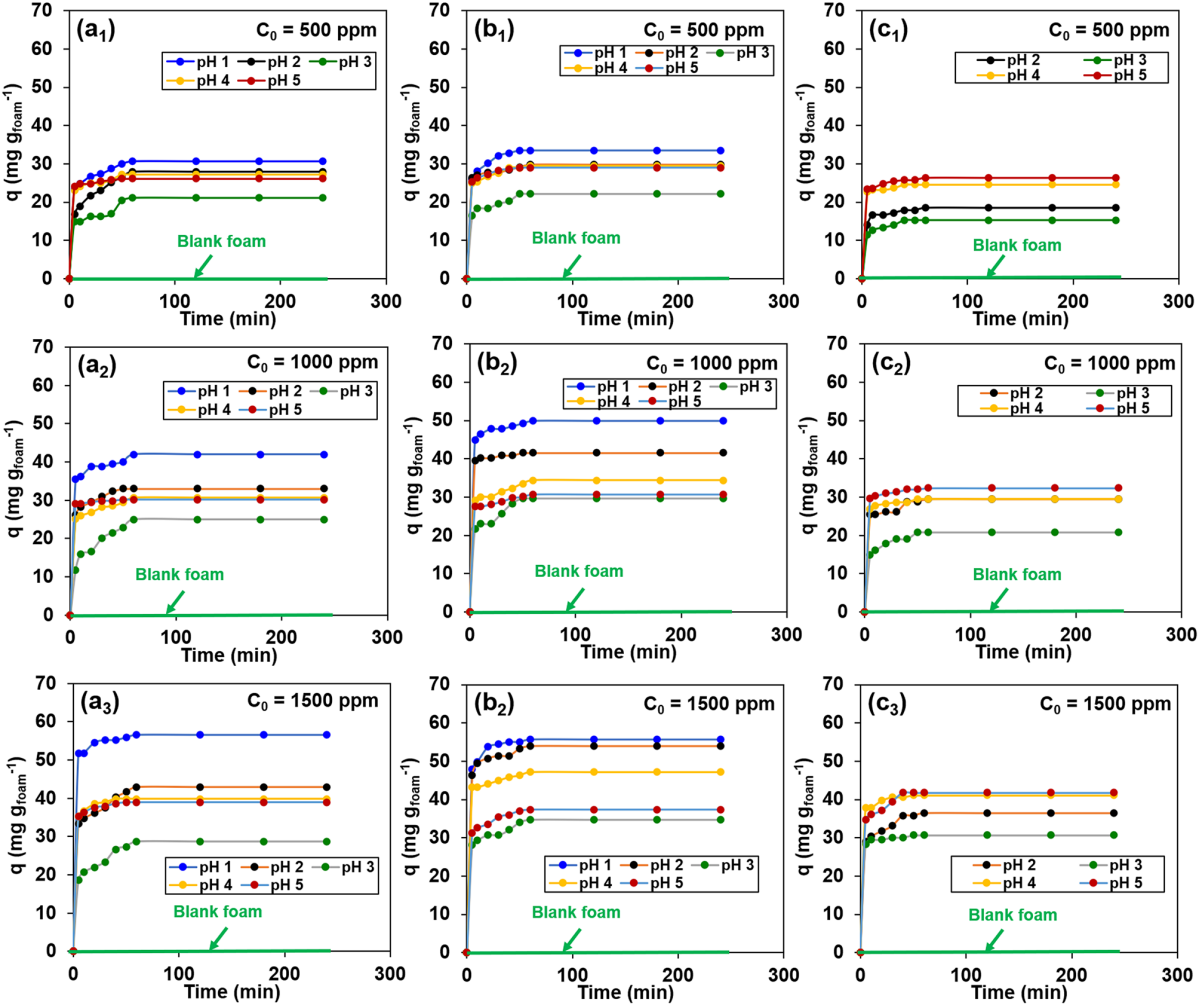
Effects of contact time, pH, and initial concentration on the functionalised carbon foam adsorption of Cu(II): (**a**_**1**_**–a**_**3**_) C-SO_3_H; **(b**_**1**_**–b**_**3**_**)** C-COOH; **(c**_**1**_**–c**_**3**_**)** C-NH_2_, and green line (—) for the blank foam (polyisoprene foam without functionalised carbon).

All carbon foams were mixed with Cu(II) solutions with various pH values and initial concentrations and monitored over the full duration of the contact time to reveal that all samples reached their equilibrium adsorption within 60 min (Fig. 4). The quantitative measurement conducted via UV–Vis analysis depends on the amount of absorbed light, which varies with metal solution concentrations for bulk analysis. From UV–Vis analysis, the blank foam specimen (polyisoprene foam without functionalised carbon) showed no change in Cu(II) solution colour for all adsorption conditions (green line in Fig. 4), the calculation of the adsorption capacity (*q*) is based on the adsorption amount of Cu(II) ion per gram of carbon foam (${\text{mg g}}_{\text{foam}}^{-1}$).

**Figure 5:**
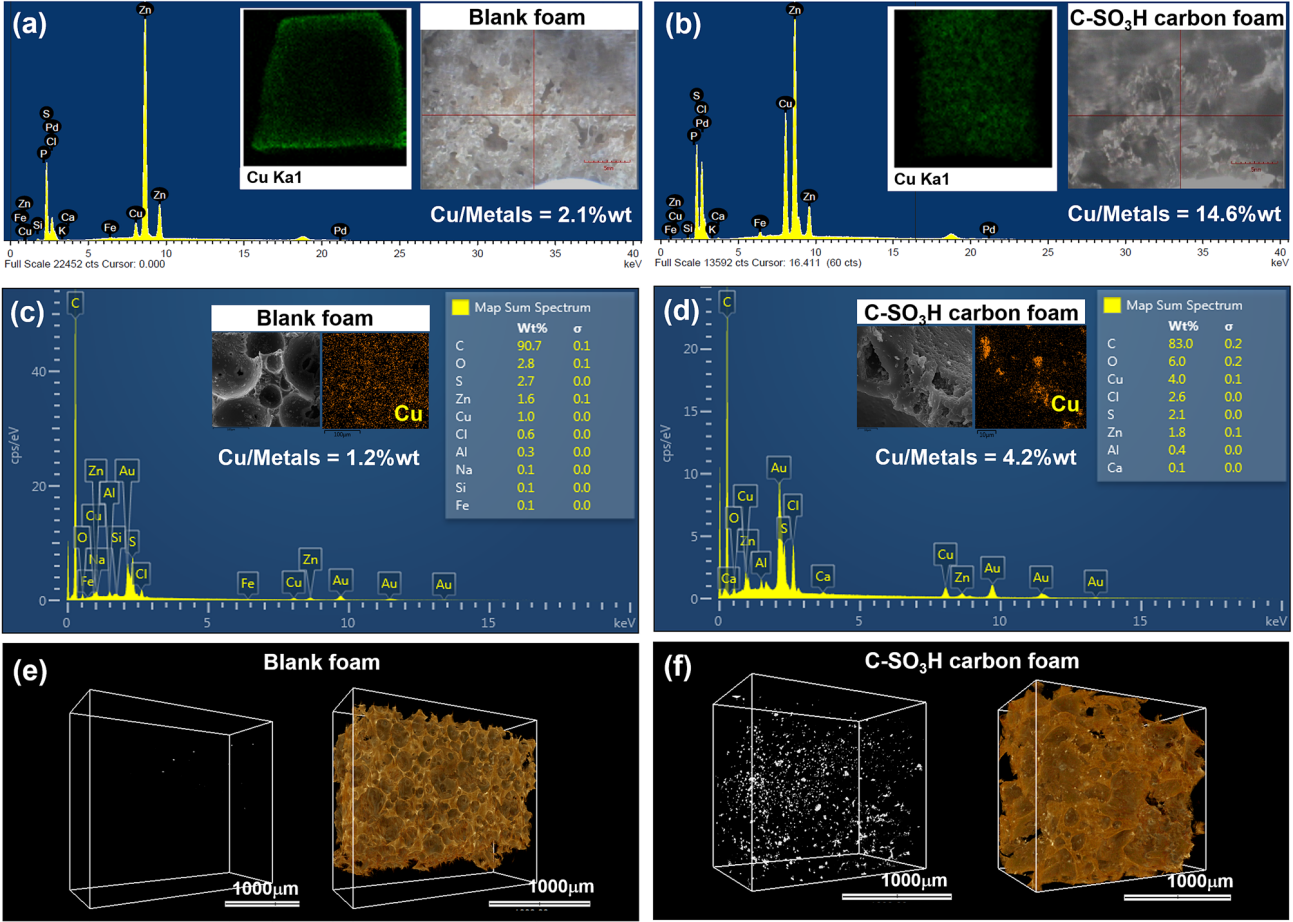
XRF **(a,b)** SEM–EDX **(c,d)** and **(e,f)** tomographic 3D visualisation of blank foam and C-SO_3_H carbon foam after Cu(II) adsorption at 1500 ppm (pH 1) for 200 min.

The used-blank and used-carbon foams were characterised by XRF and SEM-EDX to observe the amount of Cu deposition at the surface and middle of the cubic foam specimen. From Fig. 5 and Table 2, the elemental mappings of XRF, SEM-EDX, and SRXTM show a similar trend of Cu deposition, wherein the deposition at outer surface was higher than that at the centre of the foam. Moreover, the functionalised carbon foam exhibited higher Cu amount than the blank foam, especially at the outer surface (approximately 6.9 times). This can be attributed to the efficiency of metal adsorption of the functionalised carbon foam.

**Table 2:** Elemental mappings of XRF and SEM-EDX.

Foam sample	% mass of Cu/metals^a^			
	XRF		SEM–EDX	
	Outer	Middle	Outer	Middle
Blank	2.12	0.34	1.22	0.15
C-SO_3_H	14.57	4.47	4.17	0.45

^a^% mass of Cu/metals = amount of Cu × 100/(amount of metal-free Cu).

For bulk analysis, the adsorption depends on the diffusion of aqueous heavy metal ions into the open cell structure of the foam and the pores of the functionalised carbon. These ions attach to the active sites within these structures, and this mechanism requires ion-exchange and chelation. Ion-exchange is driven by electrostatic attraction between the anions of the functional groups and the cations of the heavy metal, whereas chelation is a result of the binding between the lone pairs of electrons of the functional groups and cations of the heavy metal^35^.

**Figure 6:**
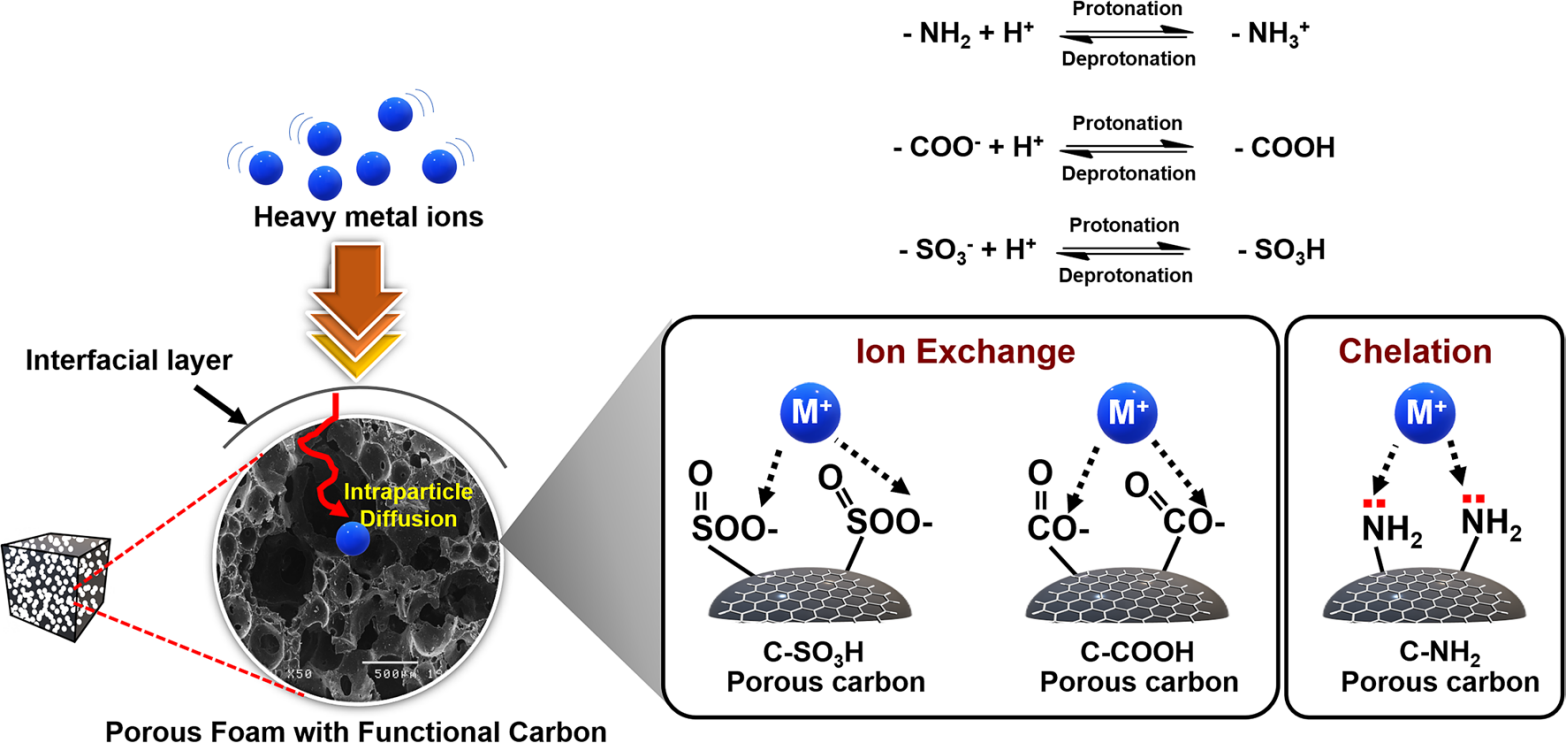
Proposed mechanism of heavy metal adsorption in the functionalised carbon foams.

The C-SO_3_H and C-COOH carbon foams adsorb Cu(II) via ion-exchange in the pH range of 1–5, with a maximum adsorption of 57 ${\text{mg g}}_{\text{foam}}^{-1}$ and 56 ${\text{mg g}}_{\text{foam}}^{-1}$, respectively, for the 1500 ppm Cu(II) solution. C-NH_2_ carbon foam adsorbs Cu(II) in a pH range of 2–5, which is mainly driven by chelation between the lone electron pairs of the –NH_2_ group and copper ions. A maximum adsorption of C-NH_2_ carbon foam was 42 ${\text{mg g}}_{\text{foam}}^{-1}$ for the 1500 ppm Cu(II) solution. However, under highly acidic conditions of pH 1, the –NH_2_ moieties are protonated to from ammonia ions (−NH_3_^+^) and are unable to adsorb the heavy metal cations. The results are consistent with the zeta potential calculations (Fig. 3c). The mechanism for heavy metal ion adsorption by the foams is shown in Fig. 6. Adsorption is not studied at pH > 5 due to metal hydroxide precipitation, which requires additional separation techniques for heavy metal removal.

According to literature review, adsorption capacity is mostly examined in batch experiments at room temperature (approx. 30 °C). Some literatures reported the temperature effect (4–90 °C) on the metal ion adsorption where the temperature has a slight effect on the adsorption of Cu(II) ions^6,36,37^. Therefore, the adsorption temperature was kept constant at 30 °C in our study.

### Adsorption kinetics of Cu(II)

The kinetic rate constant (*k*) and the correlation coefficients (*R*^*2*^) obtained from the models indicate that a pseudo-second order kinetic model best fits the adsorption mechanism (Table 3). This suggests that the adsorption mechanism involves adsorbate-adsorbent interaction and a rate-limiting step. In most cases, rate limiting is due to the chemical reactions and diffusion mechanisms. The chemical reaction involves the sharing of valence forces or exchange of lone-pair electrons from the functional groups to the metal ions, while the diffusion mechanisms include film diffusion and intraparticle diffusion^38–40^. The metal ions initially diffuse from the aqueous media to the external foam surface, from which these are transported through the carbon foam network and retained in the pore structure, where ion adsorption occurs at the active sites of the functionalised carbon.

**Table 3:** Kinetic parameters of pseudo-first order and pseudo-second order kinetic models for Cu(II) adsorption onto functionalised carbon foam.

pH	Initial concentration (ppm)	q_eq(exp)_ (${\text{mg g}}_{\text{foam}}^{-1}$)	Pseudo-first-order kinetic			Pseudo-second-order kinetic		
			k_1_ (min^-1^)	q_eq_ (${\text{mg g}}_{\text{foam}}^{-1}$)	R_1_^2^	k_2_ (g mg^-1^ min^-1^)	q_eq_ (${\text{mg g}}_{\text{foam}}^{-1}$)	R_2_^2^
**C**-**SO**_**3**_**H carbon foam**								
1	500	30.71	0.035	8.64	0.9824	0.031	29.59	0.9987
	1000	41.99	0.026	6.97	0.8860	0.023	40.16	0.9998
	1500	56.54	0.042	5.86	0.8840	0.018	56.18	0.9999
2	500	27.97	0.038	13.57	0.9846	0.035	27.03	0.9949
	1000	33.01	0.061	9.94	0.9495	0.031	33.33	0.9984
	1500	42.97	0.033	11.97	0.9140	0.022	40.98	0.9960
3	500	21.19	0.012	6.80	0.9149	0.039	17.30	0.9988
	1000	24.95	0.036	14.95	0.9604	0.039	24.15	0.9861
	1500	28.67	0.041	13.25	0.8731	0.034	27.78	0.9853
4	500	27.21	0.028	4.42	0.9258	0.035	26.11	0.9993
	1000	30.71	0.027	6.27	0.9803	0.031	29.15	0.9994
	1500	39.86	0.663	6.01	0.9770	0.025	40.49	0.9997
5	500	26.09	0.048	2.57	0.9277	0.038	25.97	0.9997
	1000	30.16	0.037	1.30	0.9185	0.033	30.03	1.0000
	1500	38.95	0.064	5.22	0.9669	0.026	39.06	0.9998
**C**-**COOH carbon foam**								
1	500	33.42	0.069	11.06	0.9891	0.031	34.25	0.9990
	1000	49.96	0.033	5.16	0.9162	0.020	49.02	0.9999
	1500	55.74	0.072	10.66	0.9784	0.018	56.50	0.9999
2	500	29.81	0.023	3.58	0.9266	0.032	28.65	0.9997
	1000	41.53	0.032	2.10	0.8766	0.024	41.15	0.9999
	1500	53.90	0.029	6.82	0.8120	0.018	52.08	1.0000
3	500	22.12	0.028	5.99	0.9289	0.043	20.83	0.9981
	1000	29.64	0.047	11.86	0.8295	0.033	29.24	0.9861
	1500	34.78	0.023	7.07	0.9266	0.027	32.57	0.9990
4	500	29.44	0.061	7.63	0.8896	0.034	29.59	0.9981
	1000	34.36	0.023	5.92	0.9260	0.028	32.57	0.9988
	1500	47.15	0.024	4.79	0.9494	0.021	46.30	0.9996
5	500	28.96	0.069	5.57	0.9891	0.035	29.24	0.9997
	1000	30.74	0.033	4.41	0.8973	0.032	30.03	0.9990
	1500	37.28	0.045	7.75	0.9764	0.027	36.90	0.9990
**C**-**NH**_**2**_** carbon foam**								
2	500	18.51	0.047	4.45	0.8829	0.054	18.35	0.9988
	1000	29.45	0.043	6.48	0.6549	0.033	28.74	0.9930
	1500	36.49	0.061	12.41	0.8310	0.028	36.63	0.9949
3	500	15.25	0.037	4.07	0.9707	0.068	15.77	0.9943
	1000	20.82	0.037	6.69	0.9343	0.046	20.12	0.9992
	1500	30.66	0.037	2.24	0.8432	0.032	30.40	0.9999
4	500	24.61	0.018	1.85	0.8151	0.041	24.69	0.9987
	1000	29.44	0.031	2.51	0.9024	0.033	28.90	1.0000
	1500	41.08	0.081	6.08	0.9514	0.024	41.32	0.9998
5	500	26.35	0.048	4.01	0.9930	0.038	26.25	0.9998
	1000	32.35	0.054	3.83	0.9340	0.031	32.26	0.9997
	1500	41.78	0.054	10.46	0.9416	0.024	42.74	0.9961

The selective adsorption of mixed metal ions is a useful information for wastewater treatment application; however, the results could not observe by using a simple UV-spectrophotometer due to the detection limit of the instrument. From literatures, the adsorption isotherm of mixed metal ion solution have been reported. For example, the adsorption isotherm of mixed metal ions solutions (Ag(I), Cu(II), Fe(II), and Pb(II)) by an aminated polyacrylonitrile nanofiber mat using ICP technique was fitted with the Langmuir adsorption isotherm. The Langmuir constants (K_L_) were in an order of Fe(II) > Cu(II) > Pb(II) > Ag(I) which related to the affinity of binding sites of each metal ions, the low K_L_ values indicate low adsorption rate of metal ion. The adsorption of Cu(II) ions in a mixture of four ion metals was achieved at 30 mg g^−1^^41^. The selective removal capability of Cu(II) and Fe(II) by chelating resin (Rexp-501) using AAS technique indicated that the adsorption of Fe(II) was depended on the resin dosage and was not influenced by the Cu(II) concentration, as the adsorption of Cu(II) was limited to a very low level^42^. Therefore, the high sample throughput analytical process by using AAS or ICP is necessary for the selective adsorption of mixed metal ions study.

### Reusability of the composites

**Figure 7:**
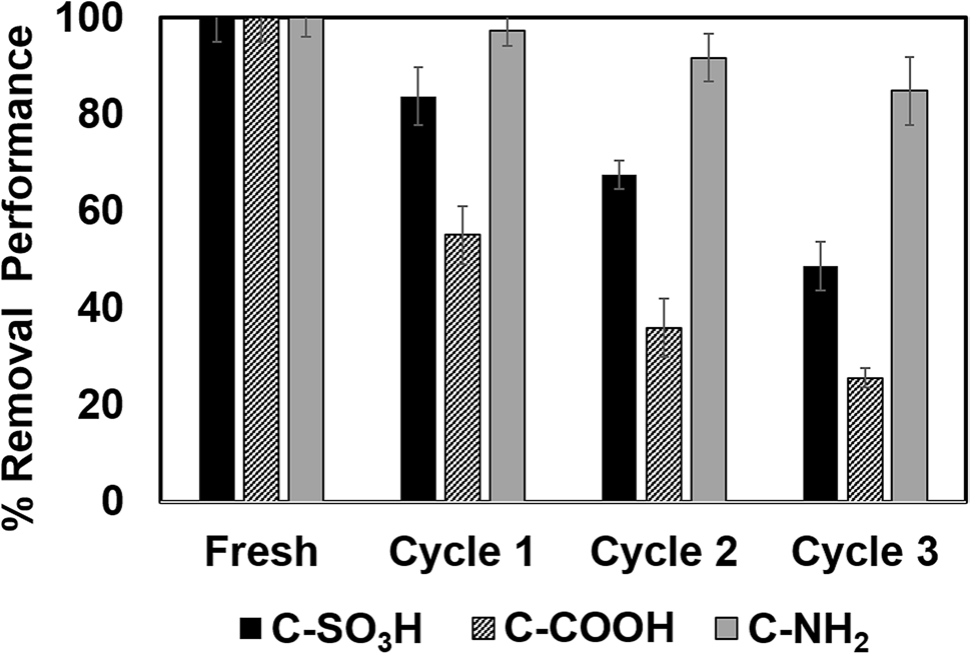
Reusability of the functionalised carbon foams.

The removal efficiencies of all the functionalised carbon foams decrease with increasing reuse cycles (Fig. 7). Desorption requires a sufficient amount of H^+^ from the desorbing agent to cover the adsorbent surface and replace the metal ions^43^; decreased removal in the third and fourth cycles is attributed to an increase in the protonated sites on the adsorbent surface^38^. The protonated groups exhibit low levels of complexation with metal ions and ion-exchange, which reduce the adsorption capacity^44^. These adsorbents are reusable, but a decreased efficiency is observed after the several cycles. The functional groups on carbon plays an important role in Cu(II) adsorption where C-SO_3_H and C-COOH carbon foams adsorb Cu(II) via ion-exchange and C-NH_2_ carbon foams adsorb Cu(II) via chelation between the lone electron pairs, a weaker interaction. In this work, the reusability of the functionalised carbon foams was studied via repeated adsorption/desorption cycles by using 0.1 M HCl as the desorbing solution and 0.1 M NaOH as the regeneration solution. An acid desorption agent is widely used as an acidic desorption agent for desorption of heavy metal ions, however, some researchers have found that acidic eluents also reduce the metal ion adsorption capabilities due to the saturation and occupation of adsorption sites with strongly adsorbed adsorbates which affect to functional groups in carbon foam by covering the active sites and reduced adsorption capacity^45,46^. Severely attenuated removal efficiencies of C-SO_3_H and C-COOH probably due to the deactivation of sulfonated carbon and carboxylated carbon via proton leaching induced by ion exchange process whilst the lesser types of interactions like physical force and complexation of C-NH_2_ might take slightly effect on the removal performance^47^.

## Conclusions

Three types of functionalised carbons (C-SO_3_H, C-COOH, C-NH_2_) were successfully incorporated into a rubber foam matrix with good distribution. UV–Vis analysis was found efficient for bulk adsorption, and the amount of Cu on the foam surface could be characterised by XRF and SEM-EDX. Sulfonyl group (−SO_3_H) foam exhibited the maximum Cu(II) adsorption of 1672 ${\text{mg g}}_{\text{active carbon}}^{-1}$, whereas the carbonyl group (−COOH) and amine group (−NH_2_) foams showed Cu(II) adsorptions of 1705 ${\text{mg g}}_{\text{active carbon}}^{-1}$ and 1293 ${\text{mg g}}_{\text{active carbon}}^{-1}$, respectively. Cu(II) adsorption was optimal in the pH range of 2–5 for all functionalised carbon foams. The adsorption mechanism revealed a pseudo-second order kinetics model. The functionalised carbon foams could be reused for up to four cycles, although a gradual decrease in removal efficiency was observed. This approach offers a bio-renewable polymer material capable of adsorbing heavy metal ions from the aqueous media and shows potential for effective wastewater treatment. The selective metal ion adsorption for mixed metal ion with proper measurements, such as atomic adsorption spectroscopy (AAS), and inductively coupled plasma spectrometer (ICP), would be beneficial to further study about the selective adsorption investigation of mixed metal ions, especially for wastewater treatment application.

## Materials and methods

### Materials

Activated carbon produced from coconut shell waste (surface area = 768 m^2^ g^-1^) was sourced from Carbokarn Co., Ltd. (Bangkok, Thailand). Acrylic acid (99%) and ammonium hydroxide were purchased from Sigma-Aldrich (St. Louis, Missouri, USA). Sulfuric acid (conc.), copper (II) acetate, and sodium hydroxide were obtained from Ajax Finechem Pty, Ltd. (Taren Point, New South Wales, Australia). Triton X-100 (Acros Organics, Geel, Belgium) was used as a surfactant. Hydrochloric acid (37%) was purchased from QRëC (Rawang, Selangor, Malaysia).

### Preparation of functionalised carbon and composite foam

Functionalisation was conducted in a Teflon-lined acid digestion vessel. Sulfonation was achieved by a hydrothermally treating a mixture of 1.0 g of carbon with 20 mL conc. H_2_SO_4_ at 170 °C for 12 h. The mixture was cooled, filtered, washed with DI water until neutralised, and further washed with ethanol. The functionalised carbon material (C-SO_3_H) was dried at 90 °C for 4 h and stored in a desiccator. The same hydrothermal protocol was used for the production of carboxyl-functionalised carbon, C-COOH (1.0 g of carbon with 2 mL of acrylic acid), and amine-functionalised carbon, C-NH_2_ (1.0 g of carbon mixed with 4 mL of conc. ammonium hydroxide). The methods reported in a previous study by the current authors^35^ were used for the preparation of a foam from natural rubber latex (NRL, *Hevea Brasiliensis*) and for the determination of its density and porosity. A carbon slurry was produced using a high energy ball mill (Emax, Retsch GmbH, Haan, Germany) containing tungsten carbide balls (5 mm in diameter; 180 balls per jar), whereby the carbon powder (8 g) was ground with Triton-X (1.4 g dissolved in 18.6 mL of deionized water) at 800 rpm for 30 min. The carbon slurry was added to high ammonia rubber latex at concentrations of 1–7 phr (parts per hundred of rubber). The density of the foam was controlled at 0.29 ± 0.1 g cm^−3^ and the porosity at 64 ± 4%. A static compression set test was performed following ASTM D395 (Method B, Type 2 at 70 °C for 24 h) to verify that the flexibility and elasticity of the foam fell within the range of 65–75%.

### Characterisation of functionalised carbon and composite foam

The surface area of the carbon powder was characterised using N_2_ isotherm (V-Sorb 2800P, Gold APP Instruments Corp., Beijing, China) through Brunauer-Emmett-Teller (BET) analysis. The morphologies of the functionalised carbon and composite foam were investigated using scanning electron microscopy (SEM, JSM-6510LV, JEOL Ltd., Tokyo, Japan) at an acceleration voltage of 20 kV. Elemental mapping of the used-functionalised carbon and used-composite foam were analysed by scanning electron microscope (SEM, FEI, Quanta 450) and energy dispersive X-ray spectrometer (EDX, Oxford, XMax) and XRF microscopy (Horiba, XGT-7200). X-ray photoelectron spectroscopy (XPS) data were recorded using an ULVAC-PHI, PHI 500 Versa Probe II (ULVAC-PHI Inc., Kanagawa, Japan) with Al Kα X-ray radiation as the excitation source. The atomic concentrations of the functionalised carbon were analysed from the C1s, O1s, N1s, and S2p spectra. The instrument sensitivity was 0.01–0.5% (atomic percentage). Tomographic volume of the samples were investigated by synchrotron radiation X-ray tomographic microscopy (SRXTM). The sample projections (10 keV, at a distance of 34 m from the source) were obtained from the detection system, which was equipped with 200-μm-thick YAG:Ce scintillator (Crytur, Czech Republic), the white-beam microscope (Optique Peter, France) and the pco.edge 5.5 sCMOS camera (2560 × 2160 pixels, 16 bits). After collecting data, it was normalised using flat-field correction algorithm and reconstructed by Octopus reconstruction, respectively. To provide the 3D representation of tomographic volume of the sample, the reconstructed images were rendered using Drishti software.

### Cu(II) adsorption

Heavy metal removal was performed in a batch system in which the adsorbent was added to the freshly prepared Cu(II) solutions with varying concentrations and pH and monitored for the duration of the contact time. Standard solutions of Cu(II) in a range of concentrations (500–1500 ppm) were prepared by dissolving copper acetate (Cu(CH_3_COO)_2_.5H_2_O) in deionised water. The pH values of each Cu(II) solution were adjusted in the pH range of 1–5 using 0.1 M HCl or 0.1 M NaOH. The adsorption was performed at 150 rpm on an orbital shaker, where the adsorbent was added to the conical tubes containing 25 ml of freshly prepared Cu(II) solutions for a total contact time of 0–250 min at a constant temperature of 303 K. Ion adsorption was characterised using UV–Visible spectrophotometer (Shimadzu UV 1700, Shimadzu Corp., Kyoto, Japan). The adsorption capacities (q) were calculated according to Eq. (1).

1 $$q = \, \left( {C_{i} {-}C_{t} } \right) \times {\text{V}}/{\text{m}},$$

where *q* is the adsorption amount (${\text{mg g}}_{\text{foam}}^{-1}$), *C*_*i*_ and *C*_*t*_ are the initial and final concentrations (mg L^-1^), respectively, *m* is the weight of active carbon (g), and *V* is the volume of the solution (L).

For the adsorption kinetics, the pseudo-first-order kinetic and pseudo-second-order kinetic equations are expressed as follows:

2 $$\mathbf{log}\left({{\varvec{q}}}_{{\varvec{e}}}-{{\varvec{q}}}_{{\varvec{t}}}\right)=\mathbf{log}{{\varvec{q}}}_{{\varvec{e}}}-\frac{{{\varvec{k}}}_{1}{\varvec{t}}}{2.303}$$

3 $$\frac{1}{{{\varvec{q}}}_{{\varvec{t}}}}=\boldsymbol{ }\frac{1}{{{\varvec{q}}}_{{\varvec{e}}}}+\boldsymbol{ }\frac{1}{{{\varvec{k}}}_{2}{{\varvec{q}}}_{{\varvec{e}}}^{2}}$$

where *q*_*t*_ and *q*_*e*_ are the amounts of adsorbed copper per weight of carbon foam (${\text{mg g}}_{\text{foam}}^{-1}$) at time *t* (min) and at equilibrium, respectively. Rate constant of pseudo-first-order adsorption is *k*_*1*_ (min^−1^) and pseudo-second-order adsorption is *k*_*2*_ (g^−1^ mg min^−1^).

The reusability of the functionalised carbon foams was studied via repeated adsorption/desorption cycles. The spent adsorbents can be recovered by acid washing adsorbents by 0.1 M HCl as the desorbing solution and 0.1 M NaOH as the regeneration solution. The regenerated carbon foams were washed with distilled water up to neutral pH and then reused in a new adsorption/desorption cycle. Four consecutive adsorption/desorption cycles were performed with three replications (n = 3).
